# Supplementary material for: Global Seroprevalence of Tick-Borne Encephalitis Antibodies in Humans, 1956–2022: A Literature Review and Meta-Analysis
Source: Vaccines (Basel). 2024 Jul 30;12(8):854. doi: 10.3390/vaccines12080854 (PMC11360530; doi:10.3390/vaccines12080854)

**Supplemental File S5.** Summary of total included articles, population group studies, and study participants for each country included for review. The minimum-maximum reported anti-TBEV seroprevalence in general population and high-risk population groups studies in each country are provided.

| Continent | Geographic Region | Country | Published Articles | General population group studies | High-risk population group studies | Total population group studies | General population participants | High-risk population participants | Total participants | Min-max general population anti-TBEV seroprevalence (%, years)[1,2] | Min-max high-risk population anti-TBEV seroprevalence (%, years)[1,2] |
|---|---|---|---|---|---|---|---|---|---|---|---|
| Africa | Asia & Africa | Djibouti | 1 | 1 | 0 | 1 | 893 | 0 | 893 | 0.11-0.11 (2010-2011) | - |
| Africa | Asia & Africa | Togo | 1 | 1 | 0 | 1 | 169 | 0 | 169 | 6.92-6.92 (NR) | - |
| Asia | Asia & Africa | Japan | 1 | 0 | 1 | 1 | 0 | 291 | 291 | - | 0.69-0.69 (2017-2017) |
| Asia | Asia & Africa | Malaysia | 1 | 0 | 1 | 1 | 0 | 85 | 85 | - | 5.88-5.88 (2012-2013) |
| Asia | Asia & Africa | Mongolia | 3 | 2 | 1 | 3 | 2717 | 993 | 3710 | 2.2-14.6 (2003-2016) | 20.2-20.2 (2016-2022) |
| Asia | Asia & Africa | South Korea | 2 | 0 | 2 | 2 | 0 | 896 | 896 | - | 0.34-1.91 (2015-2018) |
| Asia | Asia & Africa | Turkey | 8 | 6 | 2 | 8 | 4162 | 551 | 4713 | 0.0-4.54 (2002-2016) | 1.44-2.93 (2006-2007) |
| Europe | Northwestern Eurasia | Finland | 5 | 2 | 4 | 6 | 8295 | 1579 | 9874 | 0.34-0.7 (NR) | 2.4-26.4 (1958-2022) |
| Europe | Northwestern Eurasia | Lithuania | 2 | 2 | 1 | 3 | 8513 | 595 | 9108 | 3.13-6.6 (2000-2000) | 2.69-2.69 (2000-2000) |
| Europe | Northwestern Eurasia | Russia | 5 | 5 | 0 | 5 | 7073 | 0 | 7073 | 0.0-50.7 (1965-2012) | - |
| Europe | Scandinavia | Denmark | 2 | 2 | 1 | 3 | 1500 | 39 | 1539 | 0.1-5.0 (1960-2022) | 28.2-28.2 (1960-1960) |
| Europe | Scandinavia | Norway | 7 | 7 | 0 | 7 | 4747 | 0 | 4747 | 0.0-19.7 (1973-2019) | - |
| Europe | Scandinavia | Sweden | 10 | 5 | 8 | 13 | 3778 | 3773 | 7551 | 1.3-8.3 (1956-2019) | 0.0-14.1 (1987-2017) |
| Europe | Southeastern Europe | Bosnia and Herzegovina | 1 | 0 | 1 | 1 | 0 | 959 | 959 | - | 0.42-0.42 (1996-1996) |
| Europe | Southeastern Europe | Bulgaria | 1 | 1 | 0 | 1 | 1451 | 0 | 1451 | 0.62-0.62 (2015-2015) | - |
| Europe | Southeastern Europe | Croatia | 2 | 1 | 2 | 3 | 60 | 434 | 494 | 0.0-0.0 (2010-2010) | 1.44-1.44 (2000-2000) |
| Europe | Southeastern Europe | Greece | 2 | 2 | 0 | 2 | 1221 | 0 | 1221 | 2.06-4.3 (1972-2005) | - |
| Europe | Southeastern Europe | North Macedonia | 1 | 1 | 1 | 2 | 46 | 45 | 91 | 0.0-0.0 (2022-2022) | 2.22-2.22 (2022-2022) |
| Europe | Southeastern Europe | Romania | 7 | 4 | 5 | 9 | 9438 | 2711 | 12149 | 0.08-7.84 (1985-2020) | 0.67-30.1 (1972-2006) |
| Europe | Southeastern Europe | Serbia | 6 | 5 | 3 | 8 | 2305 | 614 | 2919 | 0.0-4.98 (1962-2022) | 0.0-15.04 (2019-2022) |
| Europe | Western and Central Europe | Austria | 1 | 1 | 0 | 1 | 1412 | 0 | 1412 | 15.5-15.5 (1961-1961) | - |
| Europe | Western and Central Europe | Belgium | 1 | 0 | 1 | 1 | 0 | 195 | 195 | - | 0.0-0.0 (2019-2019) |
| Europe | Western and Central Europe | Czech Republic | 9 | 9 | 2 | 11 | 3273 | 931 | 4204 | 1.05-40.3 (1959-2014) | 6.57-18.2 (1977-2018) |
| Europe | Western and Central Europe | France | 3 | 1 | 2 | 3 | 1177 | 4719 | 5896 | 0.76-0.76 (1996-1996) | 0.17-2.28 (2002-2020) |
| Europe | Western and Central Europe | Germany | 15 | 11 | 10 | 21 | 23,392 | 5349 | 28,741 | 1.4-32.8 (1865-2021) | 0.0-33.4 (1962-2013) |
| Europe | Western and Central Europe | Italy | 12 | 9 | 8 | 17 | 1521 | 1896 | 3417 | 0.0-3.6 (1965-2012) | 0.0-10 (1987-2012) |
| Europe | Western and Central Europe | Liechtenstein | 1 | 1 | 1 | 2 | 301 | 30 | 331 | 0.66-0.66 (1967-1968) | 0.0-0.0 (1967-1968) |
| Europe | Western and Central Europe | Netherlands | 2 | 1 | 2 | 3 | 356 | 868 | 1224 | 3.37-3.37 (1989-1993) | 0.53-2.56 (1989-2017) |
| Europe | Western and Central Europe | Poland | 12 | 8 | 9 | 17 | 20,299 | 24,389 | 44,688 | 0.0-8.46 (1965-2021) | 6.64-87.5 (1971-2018) |
| Europe | Western and Central Europe | Portugal | 1 | 1 | 0 | 1 | 1649 | 0 | 1649 | 0.18-0.18 (NR) | - |
| Europe | Western and Central Europe | Slovakia | 4 | 4 | 2 | 6 | 977 | 142 | 1119 | 0.18-16.1 (1959-2016) | 33.3-42.4 (1959-1964) |
| Europe | Western and Central Europe | Slovenia | 1 | 1 | 0 | 1 | 143 | 0 | 143 | 4.19-4.19 (1997-1997) | - |
| Europe | Western and Central Europe | Switzerland | 3 | 2 | 1 | 3 | 6752 | 1902 | 8654 | 1.32-5.58 (1972-2015) | 2.1-2.1 (1978-1980) |

[1]Countries were marked with "-" if no general / high-risk population group anti-TBEV seroprevalence study were previously conducted or met inclusion criteria; [2]Anti-TBEV seroprevalence among countries with only one general / high-risk population group study were labeled as both the minimum and maximum seroprevalence; NR=not reported

**Supplemental File S6.** Number of population group studies that used one, two, or three serological methods and associated serological combinations to measure anti-TBEV seroprevalence across temporal periods. The total number (A) and proportion (B) each serological method was used by population group studies are shown before 1992 or in 1992 and after. The total number of times each serological method was used in the population group studies were visualized by decade (C). Data that did not report sample collection years were excluded from total counts per decade.

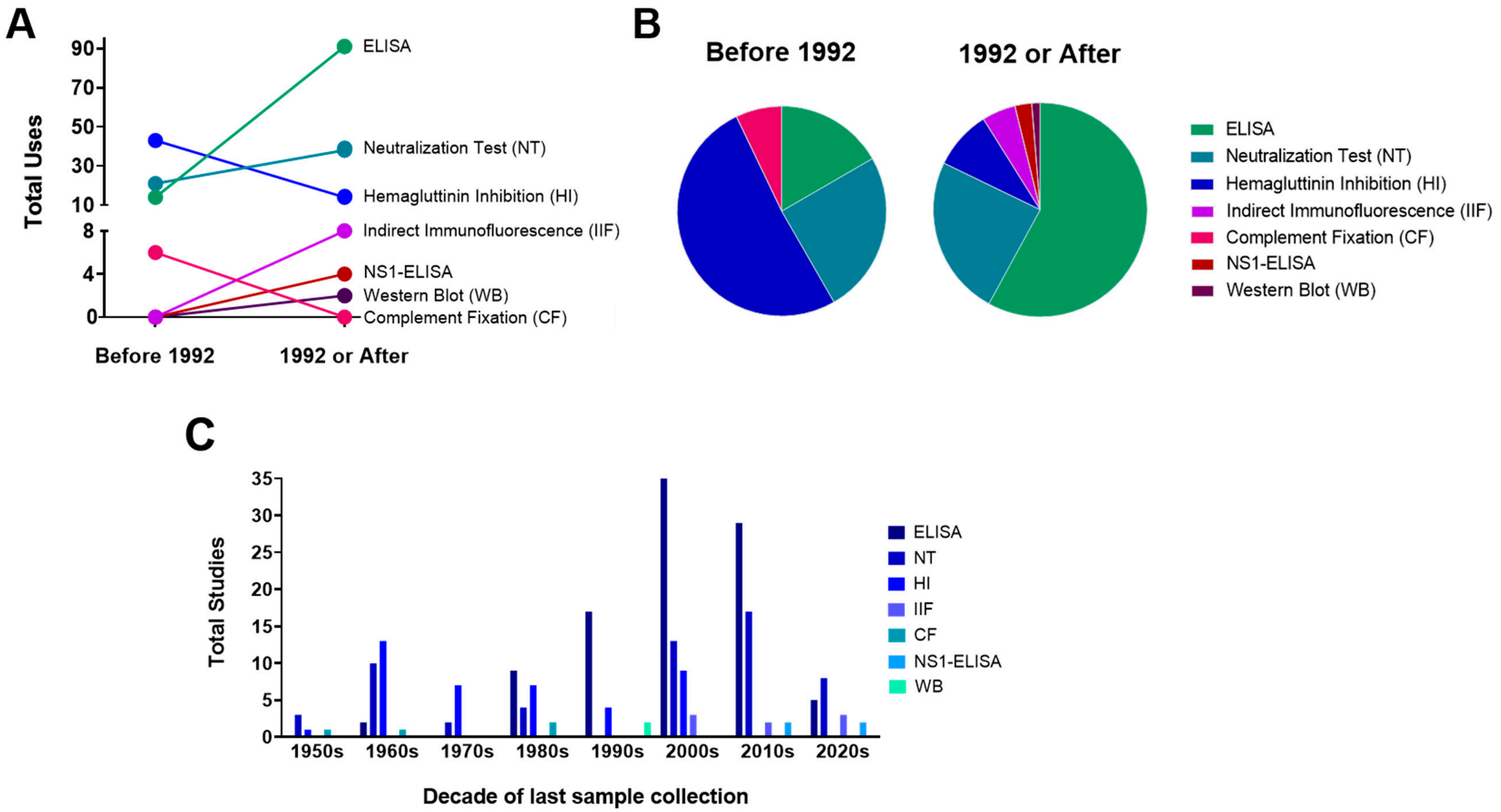

**Supplemental File S7.** Number of seroprevalence population group studies that used one, two, or three serological methods and associated serological combinations to measure anti-TBEV seroprevalence in each population group. Studies were categorized by the number of unique serological methods used to detect TBEV antibodies (one, two, or three) and the different serological combinations respective to the number of unique methods thereof are shown. The number of seroprevalence studies that measured TBEV antibodies using one, two, or three serology methods in the study participants are visualized via pie chart. No data were excluded for serological method summaries.

| **Serological Method** | **General Populations** | **High-Risk Populations** | **Total (%)*** |
|---|---|---|---|
| *Studies that used one serological method (101)* | | | |
| ELISA | 27 | 24 | 51 (50.5) |
| HI | 16 | 8 | 24 (23.8) |
| NT | 13 | 7 | 20 (19.8) |
| NS1-ELISA | 2 | 1 | 3 (3.0) |
| IIF | 1 | 1 | 2 (2.0) |
| *Studies that used two serological methods (58)* | | | |
| ELISA+NT | 13 | 12 | 25 (43.1) |
| ELISA+HI | 6 | 11 | 17 (29.3) |
| HI+NT | 4 | 2 | 6 (10.3) |
| ELISA+IIF | 2 | 1 | 3 (5.2) |
| HI+CF | 3 | 0 | 3 (5.2) |
| ELISA+WB | 2 | 0 | 2 (3.4) |
| ELISA+CF | 0 | 1 | 1 (1.7) |
| IIF+NT | 1 | 0 | 1 (1.7) |
| *Studies that used three serological methods (8)* | | | |
| ELISA+HI+NT | 2 | 3 | 5 (62.5) |
| ELISA+IIF+HI | 1 | 0 | 1 (12.5) |
| NS1-ELISA+NT+IIF | 1 | 0 | 1 (12.5) |
| NT+HI+CF | 1 | 0 | 1 (12.5) |
| ***Total (all studies)*** | ***96*** | ***71*** | ***167*** |

*Proportion (%) of each serological method within respective number of studies that used one, two, or three total methods

| **Number of serological methods used in the study** | **General Populations** | **High-Risk Populations** | **Total (%)** |
|---|---|---|---|
| One method | 60 | 41 | 101 (60.5) |
| Two methods | 31 | 27 | 58 (34.7) |
| Three methods | 5 | 3 | 8 (4.8) |
| ***Total*** | ***96*** | ***71*** | ***167*** |

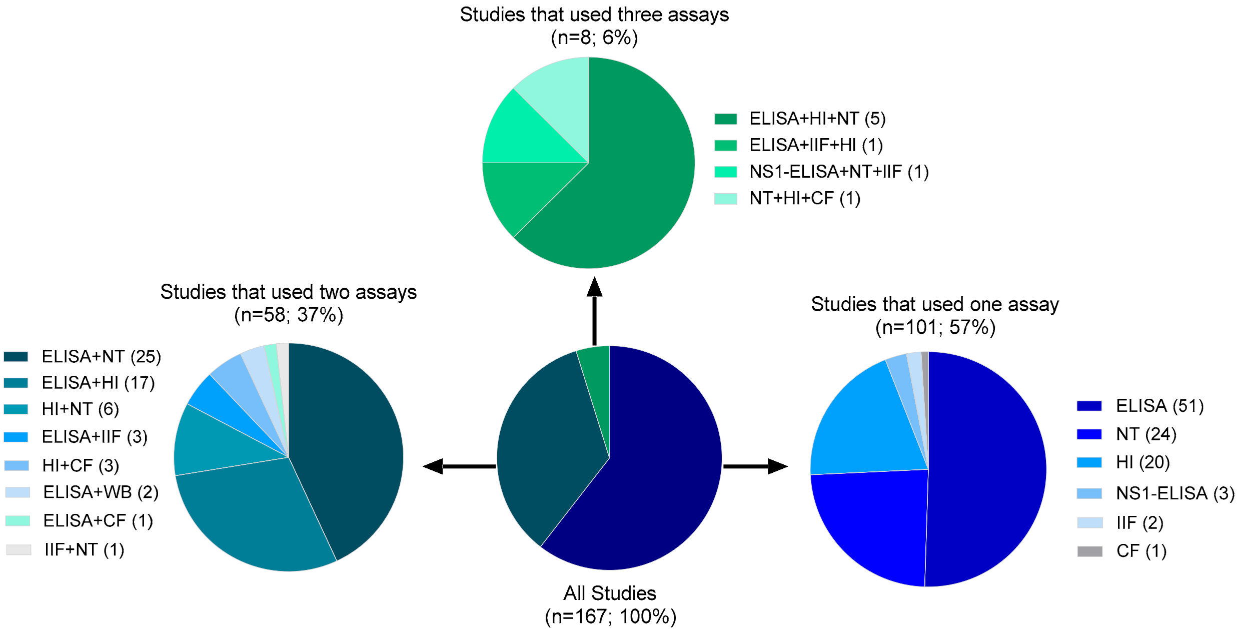
Studies that used three assays
(n=8; 6%)
ELISA+HI+NT (5)
ELISA+IIF+HI (1)
NS1-ELISA+NT+IIF (1)
NT+HI+CF (1)
Studies that used two assays
(n=58; 37%)
ELISA+NT (25)
ELISA+HI (17)
HI+NT (6)
ELISA+IIF (3)
HI+CF (3)
ELISA+WB (2)
ELISA+CF (1)
IIF+NT (1)
Studies that used one assay
(n=101; 57%)
ELISA (51)
NT (24)
HI (20)
NS1-ELISA (3)
IIF (2)
CF (1)
All Studies
(n=167; 100%)

**Supplemental File S8.** Anti-TBEV seroprevalence before 1992 or in 1992 and after within each population group across all countries, TBE endemic countries, and high-risk areas within TBE endemic countries.

| **Anti-TBEV seropositivity comparison (Sample collection before 1992 vs on or after 1992)** | | | | | |
|---|---|---|---|---|---|
| **Population Group** | **Anti-TBEV Sero-prevalence [95% CI]** | **$I^2$** | **$tau^2$** | **No. RE model observations** | **p-value** |
| General populations | | | | | |
| Before 1992 | 0.0167 [0.0124; 0.0215] | 86.3% | 0.0162 | 621 | 0.0011 |
| On or After 1992 | 0.0096 [0.0059; 0.0140] | 76.7% | 0.0072 | 241 | |
| High-risk populations | | | | | |
| Before 1992 | 0.0513 [0.0406; 0.0631] | 92.9% | 0.0143 | 133 | < 0.0001 |
| On or After 1992 | 0.0134 [0.0075; 0.0204] | 83.3% | 0.0085 | 90 | |
| General populations (Endemic)* | | | | | |
| Before 1992 | 0.0171 [0.0126; 0.0221] | 87.5% | 0.0166 | 551 | 0.4836 |
| On or after 1992 | 0.0136 [0.0092; 0.0186] | 68.4% | 0.0055 | 210 | |
| High-risk populations (Endemic)* | | | | | |
| Before 1992 | 0.0513 [0.0406; 0.0631] | 92.9% | 0.0143 | 133 | 0.0002 |
| On or after 1992 | 0.0219 [0.0118; 0.0342] | 89.5% | 0.0108 | 50 | |
| High-risk areas (Endemic)*^ | | | | | |
| Before 1992 | 0.0279 [0.0228; 0.0333] | 90.5% | 0.0166 | 601 | 0.4582 |
| On or after 1992 | 0.0270 [0.0199; 0.0349] | 76.6% | 0.0070 | 141 | |

Note: RE model based on data from studies categorized as high-quality" and "moderate-quality".
Note: For studies with sample collection years across more than one year (Y years, Y>1), the observation was split to Y observations in RE model. For each split observation, N (total subjects) in each year = N (total subjects) in Y years / Y years, n (total seropositive) in each year = n (total seropositive) in Y years / Y years.
*Country endemicity was determined at the country-level based on human TBE cases reported in the country at the time of the sample collection in the original study.
^High-risk areas were defined as samples collected or population group localities in TBE endemic areas (which are within TBE endemic countries)

**Supplemental File S9.** Random effects (RE) models of anti-TBEV seroprevalence among general and high-risk population group studies by geographic region (1956-2022). * p-value=<0.05; No data were excluded for geographic regional analyses.

| **Seropositivity of TBEV antibodies by geographic region and population group** | | | | | | | |
|---|---|---|---|---|---|---|---|
| **Geographic region** | **Population group (study participants)** | **Anti-TBEV sero-prevalence [95% CI]** | **$I^2$** | **$tau^2$** | **No. RE model observations** | **Range data collection (years)** | **p-value** |
| Western and Central Europe | General Populations (60,400) | 0.0267 [0.0210; 0.0330] | 93.7% | 0.0142 | 227 | 1959-2021 | 0.013* |
| | High-Risk Populations (40,785) | 0.0461 [0.0339; 0.0598] | 97.0% | 0.0181 | 90 | 1959-2020 | |
| Scandinavia | General Populations (10,025) | 0.0191 [0.0093; 0.0317] | 92.9% | 0.0098 | 29 | 1956-2022 | 0.248 |
| | High-Risk Populations (3812) | 0.0340 [0.0109; 0.0671] | 94.9% | 0.0188 | 15 | 1960-2017 | |
| Northwestern Eurasia | General Populations (23,881) | 0.0741 [0.0265; 0.1414] | 99.6% | 0.0430 | 15 | 1965-2012 | 0.312 |
| | High-Risk Populations (1465) | 0.1182 [0.0574; 0.1956] | 92.8% | 0.0301 | 12 | 1958-2022 | |
| Southeastern Europe | General Populations (12,726) | 0.0325 [0.0213; 0.0455] | 91.3% | 0.0109 | 51 | 1962-2022 | 0.411 |
| | High-Risk Populations (4763) | 0.0493 [0.0191; 0.0913] | 96.7% | 0.0271 | 17 | 1972-2022 | |
| Asia & Africa | General Populations (7941) | 0.0257 [0.0082; 0.0508] | 96.5% | 0.0293 | 32 | 2002-2016 | 0.668 |
| | High-Risk Populations (2816) | 0.0171 [0.0000; 0.0730] | 96.2% | 0.0369 | 14 | 2006-2022 | |

Note: Details on meta-analytical random effects model: 1. Inverse variance method; 2. DerSimonian-Laird estimator for tau^2; 3. Jackson method for confidence interval of tau^2 and tau; 4. Freeman-Tukey double arcsine transformation. No data were excluded for analyses.

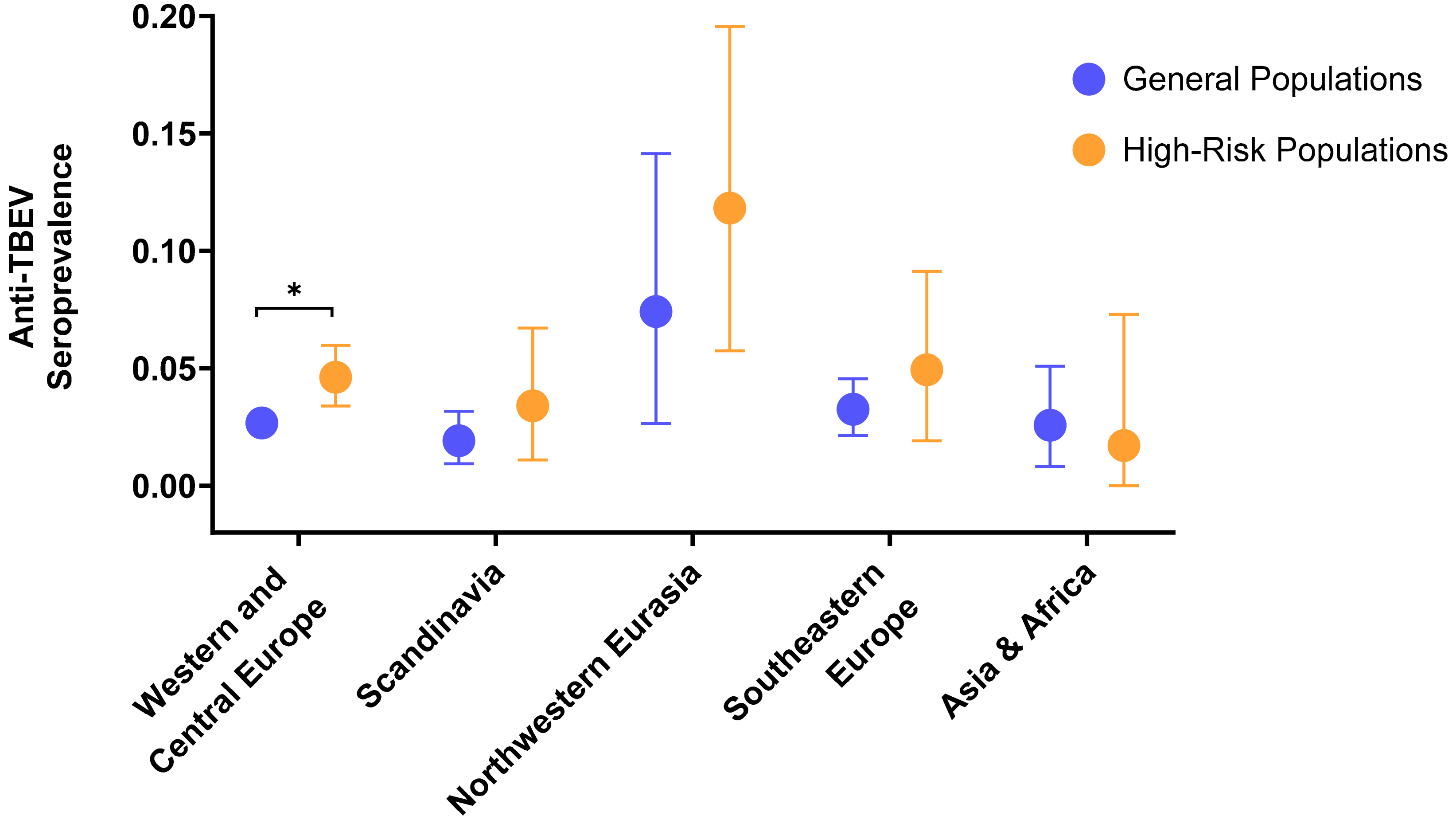

Anti-TBEV Seroprevalence
0.20
0.15
0.10
0.05
0.00
*
General Populations
High-Risk Populations
Western and Central Europe
Scandinavia
Northwestern Eurasia
Southeastern Europe
Asia & Africa

**Supplemental File S10.** Forest plot summary visualization of mean anti-TBEV seroprevalence in each population group study by geographic region: Western and Central Europe (A), Scandinavia (B), Northwestern Eurasia (C), Southeastern Europe (D), and Asia and Africa (E).

## A

| Country | Article | Population Group | Vaccination Status | Sample Collection (Years) | Serological Method(s) | Sample Size | Anti-TBEV Seroprevalence | [95% CI] |
|---|---|---|---|---|---|---|---|---|
| Austria | Groll, 1965 | General Populations | Unvaccinated | 1961 | NT+HI+CF | 1412 | 0.155 | [0.137; 0.175] |
| Belgium | Lernout, 2023 (poster) | High-Risk Populations | Unvaccinated | 2019 | ELISA+NT | 195 | 0.000 | [0.000; 0.019] |
| Czech Republic | Kolman, 1973 | General Populations | Unvaccinated | 1967-1968 | HI | 138 | 0.058 | [0.025; 0.111] |
| Czech Republic | Kriz, 2015 | General Populations | Unvaccinated | 1978-1989 | NT | 434 | 0.115 | [0.087; 0.149] |
| Czech Republic | Kriz, 2015 | General Populations | Likely unvaccinated | 2001-2014 | NT | 270 | 0.263 | [0.211; 0.320] |
| Czech Republic | Lunackova, 2003 | General Populations | Unvaccinated | 2001-2002 | ELISA | 238 | 0.155 | [0.112; 0.208] |
| Czech Republic | Ackermann-Gaumann, 2023 | High-Risk Populations | Vaccinated | 2017-2018 | NS1-ELISA | 898 | 0.066 | [0.050; 0.084] |
| Czech Republic | Hubalek, 1986 | High-Risk Populations | Unvaccinated | 1977 | NT+HI | 33 | 0.182 | [0.070; 0.355] |
| Czech Republic | Kolman, 1972 | General Populations | Unvaccinated | not described | HI+CF | 84 | 0.012 | [0.000; 0.065] |
| Czech Republic | Kopecky, 1972 | General Populations | Unvaccinated | 1963-1964 | HI | 1052 | 0.110 | [0.092; 0.131] |
| Czech Republic | Kopecky, 1972 | General Populations | Unvaccinated | 1966-1967 | HI | 154 | 0.403 | [0.324; 0.485] |
| Czech Republic | Januska, 1990 | General Populations | Unvaccinated | not described | NT | 475 | 0.011 | [0.003; 0.024] |
| Czech Republic | Nedvidek, 1962 | General Populations | Unvaccinated | 1959 | CF | 428 | 0.129 | [0.098; 0.164] |
| France | Rigaud, 2016 | High-Risk Populations | Unvaccinated | 2002-2003 | ELISA | 2941 | 0.023 | [0.018; 0.029] |
| France | Schumacher, 1999 | General Populations | Unvaccinated | 1996 | ELISA+WB | 1177 | 0.008 | [0.004; 0.014] |
| France | Septfons, 2023 | High-Risk Populations | Unvaccinated | 2019-2020 | ELISA+NT | 1778 | 0.002 | [0.000; 0.005] |
| Germany | Ackermann, 1986 | General Populations | Unvaccinated | 1978-1984 | NT | 8526 | 0.016 | [0.013; 0.019] |
| Germany | Ackermann, 1986 | High-Risk Populations | Unvaccinated | 1978-1984 | NT | 237 | 0.042 | [0.020; 0.076] |
| Germany | Euringer, 2023 | General Populations | Unvaccinated | 2021 | NS1-ELISA+NT+IIF | 2200 | 0.057 | [0.048; 0.067] |
| Germany | Hunfeld, 1998 | High-Risk Populations | Unknown | not described | ELISA | 120 | 0.133 | [0.078; 0.207] |
| Germany | Hunfeld, 1998 | General Populations | Unknown | not described | ELISA | 150 | 0.067 | [0.032; 0.119] |
| Germany | Jurke, 2015 | General Populations | Unvaccinated | 2011-2013 | ELISA+NT | 103 | 0.039 | [0.011; 0.096] |
| Germany | Jurke, 2015 | High-Risk Populations | Unvaccinated | 2011-2013 | ELISA+NT | 118 | 0.025 | [0.005; 0.073] |
| Germany | Kaiser, 1997 | General Populations | Unvaccinated | 1995 | ELISA+WB | 361 | 0.094 | [0.066; 0.129] |
| Germany | Muller, 1968 | General Populations | Unvaccinated | 1965-1967 | HI | 1682 | 0.328 | [0.306; 0.351] |
| Germany | Muller, 1968 | High-Risk Populations | Unvaccinated | 1965-1967 | HI | 686 | 0.334 | [0.299; 0.370] |
| Germany | Oehme, 2002 | High-Risk Populations | Unvaccinated | 1997-1999 | ELISA | 1896 | 0.073 | [0.061; 0.085] |
| Germany | Rieger, 2002 | General Populations | Unvaccinated | 2001 | ELISA | 445 | 0.018 | [0.008; 0.035] |
| Germany | Scheid, 1964 | General Populations | Unvaccinated | 1962 | NT | 280 | 0.014 | [0.004; 0.036] |
| Germany | Scheid, 1964 | High-Risk Populations | Unvaccinated | 1962 | NT | 546 | 0.022 | [0.011; 0.038] |
| Germany | Rieger, 1999 | High-Risk Populations | Unvaccinated | 1995-1997 | ELISA | 359 | 0.022 | [0.010; 0.043] |
| Germany | Rieger, 1999 | General Populations | Unvaccinated | 1995-1996 | ELISA | 347 | 0.020 | [0.008; 0.041] |
| Germany | Clement, 1992 | High-Risk Populations | Unvaccinated | 1989 | ELISA | 614 | 0.057 | [0.040; 0.078] |
| Germany | Suss, 1995 | General Populations | Unknown | 1980-1984 | ELISA | 4300 | 0.014 | [0.011; 0.018] |
| Germany | Muller, 1987 | High-Risk Populations | Unvaccinated | 1985-1986 | ELISA+CF | 400 | 0.000 | [0.000; 0.009] |
| Germany | Ackermann, 1968 | General Populations | Unvaccinated | 1964-1967 | NT | 4998 | 0.016 | [0.012; 0.019] |
| Germany | Ebke, 1971 | High-Risk Populations | Unvaccinated | not described | HI | 373 | 0.000 | [0.000; 0.010] |
| Italy | Balducci, 1967 | General Populations | Unvaccinated | 1965 | HI+NT | 166 | 0.036 | [0.013; 0.077] |
| Italy | Cinco, 2004 | High-Risk Populations | Unvaccinated | 2002 | ELISA+HI+NT | 174 | 0.006 | [0.000; 0.032] |
| Italy | Cristofolini, 1993 | High-Risk Populations | Not described | 1987-1992 | ELISA+HI | 465 | 0.011 | [0.004; 0.025] |
| Italy | Di Renzi, 2010 | General Populations | Not described | 2008 | ELISA+HI | 282 | 0.000 | [0.000; 0.013] |
| Italy | Di Renzi, 2010 | High-Risk Populations | Not described | 2008 | ELISA+HI | 145 | 0.000 | [0.000; 0.025] |
| Italy | Lopes, 1970 | General Populations | Unvaccinated | 1968-1969 | HI | 92 | 0.000 | [0.000; 0.039] |
| Italy | Pugliese, 2007 | High-Risk Populations | Unknown | 2001-2004 | ELISA | 70 | 0.057 | [0.016; 0.140] |
| Italy | Pugliese, 2007 | General Populations | Unknown | 1993-2004 | ELISA | 156 | 0.013 | [0.002; 0.046] |
| Italy | Remoli, 2018 | General Populations | Unknown | 2012 | ELISA+NT | 100 | 0.000 | [0.000; 0.036] |
| Italy | Remoli, 2018 | High-Risk Populations | Unknown | 2012 | ELISA+NT | 101 | 0.000 | [0.000; 0.036] |
| Italy | Tomao, 2005 | General Populations | Unvaccinated | 1999-2001 | ELISA+HI+NT | 365 | 0.000 | [0.000; 0.010] |
| Italy | Tomao, 2005 | High-Risk Populations | Unvaccinated | 1999-2001 | ELISA+HI+NT | 412 | 0.000 | [0.000; 0.009] |
| Italy | Venturi, 2009 | High-Risk Populations | Unvaccinated | 2001-2006 | ELISA+HI+NT | 479 | 0.027 | [0.015; 0.046] |
| Italy | Verani, 1967 | General Populations | Unvaccinated | 1965 | HI+NT | 205 | 0.020 | [0.005; 0.049] |
| Italy | Verani, 1977 | General Populations | Unvaccinated | 1974 | HI | 55 | 0.000 | [0.000; 0.065] |
| Italy | Pugliese, 2002 | High-Risk Populations | Unknown | not described | ELISA | 50 | 0.100 | [0.033; 0.218] |
| Italy | Pugliese, 2002 | General Populations | Unknown | not described | ELISA | 100 | 0.000 | [0.000; 0.036] |
| Liechtenstein | Krech, 1992 | General Populations | Unvaccinated | 1967-1968 | ELISA+HI | 301 | 0.007 | [0.001; 0.024] |
| Liechtenstein | Krech, 1992 | High-Risk Populations | Unvaccinated | 1967-1968 | ELISA+HI | 30 | 0.000 | [0.000; 0.116] |
| Netherlands | Hofhuis, 2021 | High-Risk Populations | Unvaccinated | 2017 | ELISA+NT | 556 | 0.005 | [0.001; 0.016] |
| Netherlands | Moll van Charante, 1998 | General Populations | Unknown | 1989 | ELISA | 356 | 0.034 | [0.018; 0.058] |
| Netherlands | Moll van Charante, 1998 | High-Risk Populations | Unknown | 1989 | ELISA | 312 | 0.026 | [0.011; 0.050] |
| Poland | Adamek, 2006 | High-Risk Populations | Unvaccinated | 2004-2005 | ELISA+HI | 51 | 0.118 | [0.044; 0.239] |
| Poland | Bojkiewicz, 2022 | General Populations | Unvaccinated | 2019-2021 | ELISA | 436 | 0.041 | [0.025; 0.064] |
| Poland | Borawski, 2020 | High-Risk Populations | Not described | 2015-2018 | ELISA | 82 | 0.073 | [0.027; 0.152] |
| Poland | Borawski, 2020 | General Populations | Not described | 2015-2018 | ELISA | 20 | 0.000 | [0.000; 0.168] |
| Poland | Cisak, 1998 | General Populations | Not described | 1994-1998 | ELISA | 130 | 0.085 | [0.043; 0.146] |
| Poland | Cisak, 1998 | High-Risk Populations | Not described | 1994-1998 | ELISA | 1583 | 0.223 | [0.203; 0.244] |
| Poland | Cisak, 2001 | High-Risk Populations | Not described | 2000-2001 | ELISA | 594 | 0.308 | [0.271; 0.347] |
| Poland | Cisak, 2003 | High-Risk Populations | Not described | 1999-2002 | ELISA | 242 | 0.174 | [0.128; 0.227] |
| Poland | Cisak, 2003 | General Populations | Not described | 1999-2002 | ELISA | 50 | 0.060 | [0.013; 0.165] |
| Poland | Prokopowicz, 1995 | General Populations | Unknown | 1993 | ELISA | 386 | 0.016 | [0.006; 0.034] |
| Poland | Prokopowicz, 1995 | High-Risk Populations | Unknown | 1993 | ELISA | 227 | 0.137 | [0.095; 0.188] |
| Poland | Stefanoff, 2008 | General Populations | Not described | 1996-2005 | ELISA+IIF+HI | 1498 | 0.023 | [0.016; 0.032] |
| Poland | Wroblewska-Mularczykowa, 1968 | General Populations | Unvaccinated | 1965-1967 | HI | 17319 | 0.019 | [0.017; 0.021] |
| Poland | Wroblewska-Mularczykowa, 1973 | High-Risk Populations | Unvaccinated | 1971-1972 | HI | 21425 | 0.066 | [0.063; 0.070] |
| Poland | Siennicka, 2010 | High-Risk Populations | Unvaccinated | 2003-2007 | ELISA | 32 | 0.875 | [0.710; 0.965] |
| Poland | Bobrowska, 1995 | High-Risk Populations | Unvaccinated | not described | ELISA | 153 | 0.203 | [0.142; 0.275] |
| Poland | Bobrowska, 1995 | General Populations | Unvaccinated | not described | ELISA | 460 | 0.013 | [0.005; 0.028] |
| Portugal | Filipe, 1974 | General Populations | Unvaccinated | not described | HI | 1649 | 0.002 | [0.000; 0.005] |
| Slovakia | Busova, 2018 | General Populations | Unvaccinated | 2014-2016 | ELISA | 428 | 0.028 | [0.015; 0.048] |
| Slovakia | Gresikova, 1967a | High-Risk Populations | Unvaccinated | 1964 | NT | 85 | 0.424 | [0.317; 0.536] |
| Slovakia | Gresikova, 1967a | General Populations | Unvaccinated | 1964 | NT | 118 | 0.161 | [0.100; 0.240] |
| Slovakia | Libikova, 1960 | General Populations | Unvaccinated | 1959 | NT | 134 | 0.112 | [0.064; 0.178] |
| Slovakia | Libikova, 1960 | High-Risk Populations | Unvaccinated | 1959 | NT | 57 | 0.333 | [0.214; 0.471] |
| Slovakia | Gresikova, 1967b | General Populations | Unvaccinated | not described | HI | 297 | 0.121 | [0.086; 0.164] |
| Slovenia | Cizman, 2000 | General Populations | Unvaccinated | 1997 | ELISA | 143 | 0.042 | [0.016; 0.089] |
| Switzerland | Ackermann-Gaumann, 2022 | General Populations | Unvaccinated | 2014-2015 | ELISA | 6223 | 0.056 | [0.050; 0.062] |
| Switzerland | Matile, 1981 | High-Risk Populations | Unknown | 1978-1980 | ELISA+HI | 1902 | 0.021 | [0.015; 0.029] |
| Switzerland | Wyler, 1973 | General Populations | Unvaccinated | 1972 | NT | 529 | 0.013 | [0.005; 0.027] |
| **Random effects model** | | | | | | **101673** | **0.052** | **[0.041; 0.065]** |

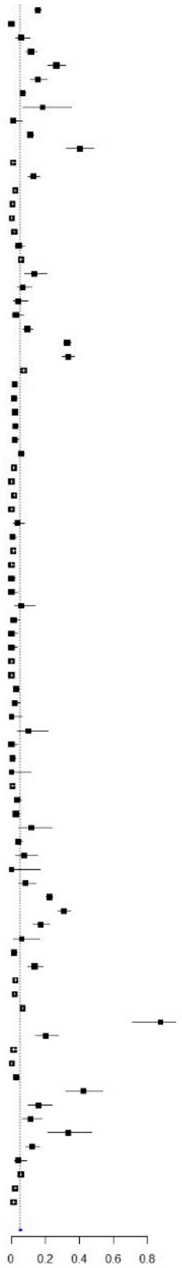

**B**

| Country | Article | Population Group | Vaccination Status | Sample Collection (Years) | Serological Method(s) | Sample Size | Anti-TBEV Seroprevalence | [95% CI] |
|---|---|---|---|---|---|---|---|---|
| Denmark | Hansen, 2023 (poster) | General Populations | Unvaccinated | 2022 | NS1-ELISA | 1000 | 0.001 | [0.000; 0.006] |
| Denmark | Freundt, 1962 | High-Risk Populations | Unvaccinated | 1960 | HI+NT | 39 | 0.282 | [0.150; 0.449] |
| Denmark | Freundt, 1962 | General Populations | Unvaccinated | 1960 | HI+NT | 500 | 0.050 | [0.033; 0.073] |
| Norway | Hjetland, 2015 | General Populations | Unvaccinated | 2010 | ELISA+NT | 1213 | 0.000 | [0.000; 0.003] |
| Norway | Larsen, 2014 | General Populations | Unvaccinated | 2012 | ELISA+NT | 461 | 0.007 | [0.001; 0.019] |
| Norway | Marvik, 2021 | General Populations | Unvaccinated | 2019 | ELISA+NT | 1023 | 0.004 | [0.001; 0.010] |
| Norway | Skarpaas, 2002 | General Populations | Unvaccinated | 1997-2000 | ELISA+NT | 126 | 0.024 | [0.005; 0.068] |
| Norway | Thortveit, 2020a | General Populations | Unvaccinated | 2016 | ELISA | 419 | 0.014 | [0.005; 0.031] |
| Norway | Thortveit, 2020b | General Populations | Unknown | 2015-2016 | ELISA | 1164 | 0.032 | [0.022; 0.044] |
| Norway | Traavik, 1979 | General Populations | Unvaccinated | 1973-1974 | HI | 341 | 0.196 | [0.156; 0.243] |
| Sweden | Gustafson, 1990 | General Populations | Unvaccinated | 1987 | ELISA+HI | 200 | 0.040 | [0.017; 0.077] |
| Sweden | Gustafson, 1990 | High-Risk Populations | Unvaccinated | 1987 | ELISA+HI | 346 | 0.116 | [0.084; 0.154] |
| Sweden | Gustafson, 1992 | High-Risk Populations | Unvaccinated | 1988-1989 | ELISA+HI | 469 | 0.017 | [0.007; 0.033] |
| Sweden | Gustafson, 1993a | General Populations | Unvaccinated | 1989 | ELISA+HI | 312 | 0.083 | [0.055; 0.120] |
| Sweden | Gustafson, 1993a | High-Risk Populations | Unvaccinated | 1989 | ELISA+HI | 433 | 0.141 | [0.110; 0.177] |
| Sweden | Gustafson, 1993b | High-Risk Populations | Unvaccinated | 1990 | ELISA+HI | 362 | 0.011 | [0.003; 0.028] |
| Sweden | Johan, 2006 | High-Risk Populations | Unvaccinated | 1998-2001 | ELISA+NT | 393 | 0.008 | [0.002; 0.022] |
| Sweden | Stjernberg, 2008 | High-Risk Populations | Unvaccinated | 1991-2002 | ELISA+NT | 400 | 0.007 | [0.002; 0.022] |
| Sweden | Svensson, 2021 | High-Risk Populations | Unvaccinated | 2017 | ELISA+NT | 99 | 0.020 | [0.002; 0.071] |
| Sweden | Svensson, 2021 | General Populations | Unknown | 2017 | ELISA+NT | 491 | 0.055 | [0.037; 0.079] |
| Sweden | Lindblom, 2014 | High-Risk Populations | Unvaccinated | 2008-2009 | ELISA+NT | 1271 | 0.002 | [0.000; 0.006] |
| Sweden | Svedmyr, 1959 | General Populations | Unvaccinated | 1956 | NT | 75 | 0.013 | [0.000; 0.072] |
| Sweden | Albinsson, 2024 | General Populations | Vaccinated | 2018-2019 | NS1-ELISA | 2700 | 0.024 | [0.018; 0.030] |
| **Random effects model** | | | | | | **13837** | **0.031** | **[0.017; 0.049]** |

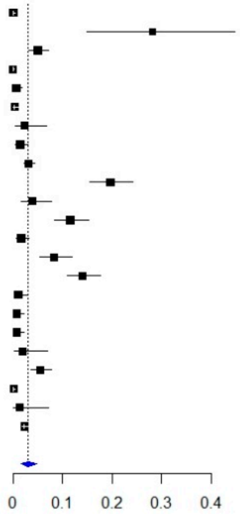


**C**

| Country | Article | Population Group | Vaccination Status | Sample Collection (Years) | Serological Method(s) | Sample Size | Anti-TBEV Seroprevalence | [95% CI] |
|---|---|---|---|---|---|---|---|---|
| Finland | Berthen, 2023 | High-Risk Populations | Vaccinated | 2018-2022 | ELISA | 100 | 0.040 | [0.011; 0.099] |
| Finland | Lindblom, 2014 | High-Risk Populations | Unvaccinated | 2008-2009 | ELISA+NT | 615 | 0.000 | [0.000; 0.006] |
| Finland | Salminen, 1961 | High-Risk Populations | Unvaccinated | 1958-1959 | HI | 716 | 0.131 | [0.107; 0.158] |
| Finland | Kroneld, 1989 | High-Risk Populations | Unknown | not described | ELISA+HI | 148 | 0.264 | [0.195; 0.342] |
| Finland | Kroneld, 1989 | General Populations | Unknown | not described | ELISA+HI | 285 | 0.007 | [0.001; 0.025] |
| Finland | Kääriäinen, 1965 | General Populations | Unvaccinated | not described | HI+NT | 8010 | 0.003 | [0.002; 0.005] |
| Lithuania | Juceviciene, 2001 | High-Risk Populations | Unvaccinated | 2000 | HI | 595 | 0.027 | [0.015; 0.043] |
| Lithuania | Juceviciene, 2001 | General Populations | Unvaccinated | 2000 | HI | 893 | 0.031 | [0.021; 0.045] |
| Lithuania | Moteiunas, 1973 | General Populations | Unvaccinated | not described | HI | 7620 | 0.066 | [0.061; 0.072] |
| Russia | Kuzyaev, 1992 | General Populations | Unvaccinated | 1966-1989 | NT | 4284 | 0.224 | [0.211; 0.237] |
| Russia | Magnaval, 2011 | General Populations | Unknown | 2007 | ELISA | 90 | 0.033 | [0.007; 0.094] |
| Russia | Magnaval, 2016 | General Populations | Unknown | 2012 | ELISA | 77 | 0.000 | [0.000; 0.047] |
| Russia | Vorontsova, 1985 | General Populations | Unvaccinated | 1965-1975 | HI | 558 | 0.507 | [0.465; 0.549] |
| Russia | L'vov, 1990 | General Populations | Unvaccinated | 1986-1987 | HI+CF | 2064 | 0.030 | [0.023; 0.038] |
| **Random effects model** | | | | | | **26055** | **0.064** | **[0.022; 0.125]** |

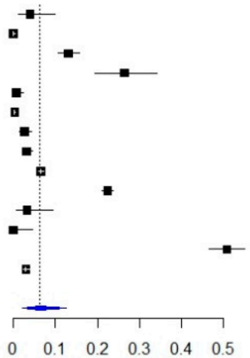

**D**

| Country | Article | Population Group | Vaccination Status | Sample Collection (Years) | Serological Method(s) | Sample Size | Anti-TBEV Seroprevalence | [95% CI] |
|---|---|---|---|---|---|---|---|---|
| Bosnia and Herzegovina | Sanchez, 2001 | High-Risk Populations | Unvaccinated | 1996 | ELISA | 959 | 0.004 | [0.001; 0.011] |
| Bulgaria | Christova, 2017 | General Populations | Unvaccinated | 2015 | ELISA+NT | 1451 | 0.006 | [0.003; 0.012] |
| Croatia | Borcic, 2001 | High-Risk Populations | Not described | 2000 | ELISA+IIF | 139 | 0.014 | [0.002; 0.051] |
| Croatia | Miletic-Medved, 2011 | General Populations | Unvaccinated | 2010 | ELISA | 60 | 0.000 | [0.000; 0.060] |
| Croatia | Miletic-Medved, 2011 | High-Risk Populations | Unvaccinated | 2010 | ELISA | 295 | 0.044 | [0.024; 0.074] |
| Greece | Pavlidou, 2008 | General Populations | Unvaccinated | 2003-2005 | ELISA+IIF | 921 | 0.021 | [0.012; 0.032] |
| Greece | Papapanagiotou, 1974 | General Populations | Unvaccinated | 1972 | HI | 300 | 0.043 | [0.023; 0.073] |
| North Macedonia | Jakimovski, 2023 | High-Risk Populations | Unvaccinated | 2022 | NT | 45 | 0.022 | [0.001; 0.118] |
| North Macedonia | Jakimovski, 2023 | General Populations | Unvaccinated | 2022 | NT | 46 | 0.000 | [0.000; 0.077] |
| Romania | Coroian, 2022 | General Populations | Unvaccinated | 2019-2020 | ELISA+NT | 1200 | 0.001 | [0.000; 0.005] |
| Romania | Ionescu, 2008 | General Populations | Not described | 1985-1993 | ELISA+HI | 5063 | 0.066 | [0.059; 0.073] |
| Romania | Ionescu, 2008 | High-Risk Populations | Not described | 1985-1993 | ELISA+HI | 209 | 0.301 | [0.240; 0.369] |
| Romania | Molnar, 2008 | General Populations | Unvaccinated | 1999-2006 | ELISA | 1669 | 0.006 | [0.003; 0.011] |
| Romania | Molnar, 2008 | High-Risk Populations | Unvaccinated | 1999-2006 | ELISA | 308 | 0.123 | [0.089; 0.165] |
| Romania | Drăgănescu, 1973 | High-Risk Populations | Unvaccinated | 1972 | HI | 1202 | 0.007 | [0.003; 0.013] |
| Romania | Drăgănescu, 1978 | General Populations | Unvaccinated | not described | HI+CF | 1506 | 0.078 | [0.065; 0.093] |
| Romania | Drăgănescu, 1975 | High-Risk Populations | Unvaccinated | not described | HI | 481 | 0.119 | [0.091; 0.151] |
| Romania | Girjabu, 1985 | High-Risk Populations | Unvaccinated | not described | HI | 511 | 0.115 | [0.089; 0.146] |
| Serbia | Banovic, 2021 | General Populations | Unknown | 2020 | IIF | 50 | 0.040 | [0.005; 0.137] |
| Serbia | Banovic, 2021 | High-Risk Populations | Unvaccinated | 2020 | IIF | 113 | 0.150 | [0.090; 0.230] |
| Serbia | Potkonjack, 2017 | General Populations | Unvaccinated | 2014-2015 | ELISA | 267 | 0.037 | [0.018; 0.068] |
| Serbia | Venturi, 2011 | General Populations | Unknown | 2005 | ELISA+HI+NT | 200 | 0.000 | [0.000; 0.018] |
| Serbia | Bordoski, 1972 | General Populations | Unvaccinated | 1962-1969 | HI | 1726 | 0.050 | [0.040; 0.061] |
| Serbia | Jakimovski, 2023 | High-Risk Populations | Unvaccinated | 2022 | NT | 51 | 0.000 | [0.000; 0.070] |
| Serbia | Jakimovski, 2023 | General Populations | Unvaccinated | 2022 | NT | 62 | 0.000 | [0.000; 0.058] |
| Serbia | Banovic, 2023 | High-Risk Populations | Unvaccinated | 2019-2021 | NT | 450 | 0.007 | [0.001; 0.019] |
| **Random effects model** | | | | | | **19284** | **0.033** | **[0.018; 0.051]** |

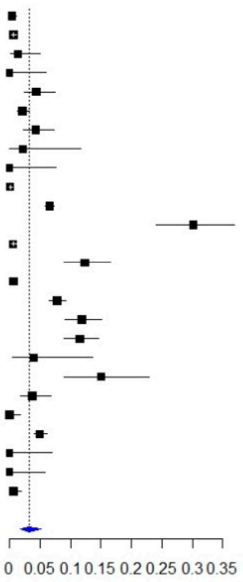

**E**

| Country | Article | Population Group | Vaccination Status | Sample Collection (Years) | Serological Method(s) | Sample Size | Anti-TBEV Seroprevalence | [95% CI] |
|---|---|---|---|---|---|---|---|---|
| Djibouti | Andayi, 2014 | General Populations | Unvaccinated | 2010-2011 | ELISA+NT | 893 | 0.001 | [0.000; 0.006] |
| Japan | Yoshii, 2017 | High-Risk Populations | Unvaccinated | 2017 | ELISA+NT | 291 | 0.007 | [0.001; 0.025] |
| Malaysia | Mohd-Shukri, 2015 | High-Risk Populations | Unvaccinated | 2012-2013 | ELISA | 85 | 0.059 | [0.019; 0.132] |
| Mongolia | Walder, 2006 | General Populations | Unvaccinated | 2003 | ELISA+NT | 545 | 0.022 | [0.011; 0.038] |
| Mongolia | Ganbold, 2023 | High-Risk Populations | Unvaccinated | 2016-2022 | ELISA | 993 | 0.202 | [0.178; 0.229] |
| Mongolia | Baasandavga, 2017 | General Populations | Unknown | 2005-2016 | ELISA+IIF | 2172 | 0.146 | [0.132; 0.162] |
| South Korea | Noh, 2021 | High-Risk Populations | Unvaccinated | 2017-2018 | ELISA+NT | 583 | 0.003 | [0.000; 0.012] |
| South Korea | Yoo, 2020 | High-Risk Populations | Unknown | 2015-2018 | ELISA | 313 | 0.019 | [0.007; 0.041] |
| Togo | Ebke, 1971 | General Populations | Unvaccinated | not described | HI | 169 | 0.059 | [0.029; 0.106] |
| Turkey | Basbulut, 2012 | General Populations | Not described | 2006 | ELISA+NT | 419 | 0.000 | [0.000; 0.009] |
| Turkey | Ergunay, 2007 | General Populations | Unknown | 2005 | ELISA | 220 | 0.036 | [0.016; 0.070] |
| Turkey | Ergunay, 2011 | General Populations | Unvaccinated | 2009 | ELISA+NT | 2454 | 0.000 | [0.000; 0.002] |
| Turkey | Esen, 2008 | General Populations | Not described | 2002-2003 | ELISA | 39 | 0.026 | [0.001; 0.135] |
| Turkey | Gunes, 2010 | High-Risk Populations | Unknown | 2006-2007 | ELISA | 273 | 0.029 | [0.013; 0.057] |
| Turkey | Tezcan, 2014 | General Populations | Unknown | 2010-2011 | IIF+NT | 920 | 0.000 | [0.000; 0.004] |
| Turkey | Uhar, 2007 | High-Risk Populations | Unvaccinated | 2006 | ELISA | 278 | 0.014 | [0.004; 0.036] |
| Turkey | Yilmaz, 2019 | General Populations | Unvaccinated | 2013-2016 | ELISA | 110 | 0.045 | [0.015; 0.103] |
| **Random effects model** | | | | | | **10757** | **0.025** | **[0.004; 0.061]** |

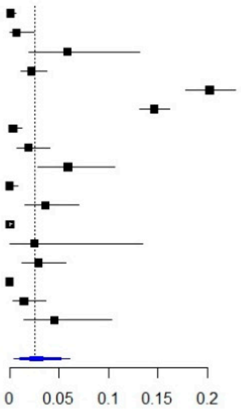

Supplement: Supplementary file 1 [file vaccines-12-00854-s001.zip › Supplemental_files_S5-S10-new_11JUN2024.pdf]
