# Supplementary material for: Global Seroprevalence of Tick-Borne Encephalitis Antibodies in Humans, 1956–2022: A Literature Review and Meta-Analysis
Source: Vaccines (Basel). 2024 Jul 30;12(8):854. doi: 10.3390/vaccines12080854 (PMC11360530; doi:10.3390/vaccines12080854)
Supplement: Supplementary file 1 [file vaccines-12-00854-s001.zip › Supplemental_File_S1.pdf]

Supplemental File S1. Exhaustive search string criteria for search strings in PubMed and Embase between 01 January 1900 and 30 June 2023 ("Search String A") and in PubMed between 01 January 1990 and 31 December 2023 ("search string two") for the systematic literature review of the global anti-TBEV seroprevalence in humans.

Search String A

Databases: PubMed; Embase
Dates: 01 January 1900 – 30 June 2023

Search terms for TBEv:

- ("Tick-borne encephalitis" [Title/Abstract] OR "tick borne encephalitis" [Title/Abstract] OR "tick-borne encephalitis virus" [Title/Abstract] OR "tick borne encephalitis virus" [Title/Abstract] OR "TBE" [Title/Abstract] OR "TBEV" [Title/Abstract] OR "Encephalitis, Tick-Borne"[Mesh] OR "Encephalitis Viruses, Tick-Borne"[MeSH] OR "Tick borne encephalit*" [Title/abstract] OR "tickborne encephalit*"[Title/Abstract] OR "tick encephalit*"[Title/Abstract] OR "tick borne meningoencephalitis"[Title/Abstract] OR "tickborne meningoencephalitis"[Title/Abstract] OR "tick-borne virus encephalit*"[Title/Abstract] OR "tickborne virus encephalit*"[Title/Abstract] OR "TBE virus*"[Title/abstract] OR TBEV[Title/Abstract] OR "Encephalitis Viruses, Tick-Borne" [Title/Abstract] OR "Encephalitis Viruses, Tick Borne" [Title/Abstract] OR "Encephalitis Virus, Tick-Borne"[Title/Abstract] OR "Encephalitis Virus, Tick Borne"[Title/Abstract] OR "Viruses, Tick-Borne Encephalitis"[Title/Abstract] OR "central european encephalit*"[Title/Abstract] OR "tick-borne viral encephalitis"[Title/Abstract] OR "tickborne viral encephalitis"[Title/Abstract] OR "tick-born encephalit*"[Title/Abstract] OR "Far Eastern Russian Encephalit*"[Title/Abstract] OR "Russian Spring-Summer Encephalit*"[Title/Abstract] OR "Louping Ill Encephalit*"[Title/Abstract] OR "Powassan Encephalit*"[Text Word] OR "Powassan Virus Diseas*"[Title/Abstract] OR "FSME" [Title/Abstract] OR "Frühsommer Meningoencephalitis" [Title/Abstract])

Search terms for Seroprevalence:

- AND – ("seroprevalence" [Title/Abstract] OR "human seroprevalence" [Title/Abstract] OR "immunity" [Title/Abstract] OR "seroprevalence immunity" [Title/Abstract] OR "natural immunity" [Title/Abstract] OR "vaccine immunity" [Title/Abstract] OR "vaccination immunity" [Title/Abstract] OR "natural vs vaccine immunity" [Title/Abstract] OR "natural vs. vaccine immunity" [Title/Abstract] OR "natural versus vaccine immunity" [Title/Abstract] OR "seropersistence" [Title/Abstract])
- OR/AND - ("antibody" [Title/Abstract] OR "antibody response" [Title/Abstract] OR "antibodies" [Title/Abstract] OR "duration" [Title/Abstract] OR "antibody duration" [Title/Abstract] OR "serology" [Title/Abstract] OR "persistence" [Title/Abstract] OR "antibody persistence" [Title/Abstract] OR "waning" [Title/Abstract] OR "waning antibody" [Title/Abstract] OR "waning antibody response" [Title/Abstract])
- OR/AND - ("Seroepidemiologic Studies"[Mesh] OR Seroepidemiolog*[Title/Abstract] OR "seroprevalen*"[Title/Abstract] OR "sero-prevalen*"[Title/Abstract] OR "serologic prevalen*"[Title/Abstract] OR "serological prevalen*"[Title/Abstract] OR "serology prevalen*"[Title/Abstract] OR "seropositiv*"[Title/Abstract] OR

"Seroconversion"[Mesh] OR seroconversion*[Title/Abstract] OR “sero-conversion*”[Title/Abstract] OR "serosurvey*"[Title/Abstract] OR “sero survey*”[Title/Abstract] OR "Antibodies"[Mesh:NoExp] OR "antibod*"[Title/Abstract] OR “immune factor*”[Title/Abstract])

Study types:

- AND (“Observational Study”[Publication Type] OR “Cohort Studies”[Mesh] OR “Longitudinal Studies”[Mesh] OR “Follow-Up Studies”[Mesh] OR “Evaluation Study”[Publication Type] OR “Cross-Sectional Studies”[Mesh] OR “Retrospective Studies”[Mesh] OR “Controlled Clinical Trials as Topic”[Mesh] OR “Registries”[Mesh] OR “Case-Control Studies”[Mesh] OR cohort*[Text Word] OR longitudinal*[Text Word] OR “follow up”[Text Word] OR evaluation[Text Word] OR “cross sectional*”[Text Word] OR “non random*”[Text Word] OR nonrandom*[Text Word] OR observation*[Text Word] OR retrospective[Text Word] OR “phase iv”[Text Word] OR “phase four”[Text Word] OR “phase 4”[Text Word] OR registr*[Text Word])

Article type:

- OR (“Review Literature as Topic”[Mesh] OR “Systematic Reviews as Topic”[Mesh] OR “Meta-Analysis as Topic”[Mesh] OR “Technology Assessment, Biomedical”[Mesh] OR systematic[sb] OR “systematic review*”[Title/Abstract] OR “systematic literature review*”[Title/Abstract] OR SLR[Title/Abstract] OR metaanalys*[Title/Abstract] OR “meta-analys*”[Title/Abstract] OR NMA[Title/Abstract] OR “technology assessment*”[Title/Abstract] OR “Systematic Review”[Publication Type] OR “Review”[Publication Type])

Exclusions:

- NOT “Comment”[Publication Type] OR “Letter”[Publication Type] OR “Editorial”[Publication Type] OR “Case Reports”[Publication Type] OR “case report*”[Title] OR “case stud*”[Title] OR “case series”[Title] OR “case histor*”[Title] OR “randomized"[Title] OR phase*[Title] OR “mice”[Title/abstract] OR “animal”[Title/abstract] OR “Flocks”[Title/abstract] OR “dogs”[Title/abstract] OR “cervids”[Title/abstract] OR “cows”[Title/abstract] OR “goat”[Title/abstract] OR “horses”[Title/abstract] OR “sheep”[Title/abstract] OR “monkeys”[Title/abstract] OR “deer”[Title/abstract] OR “equids”[Title/abstract] OR “voles”[Title/abstract] OR “bison”[Title/abstract] OR “mouse”[Title/abstract])

Text MUST include: tick borne encephalitis *OR* tick-borne encephalitis *OR* TBE - *AND* - seroprevalence *OR* antibody

Years: All – compare first search string with this search. Exclude duplicates. Identify any new studies.

Search String B

Databases: PubMed
Dates: 01 January 1900 – 31 December 2023

Search terms for TBEv:

- (“Tick-borne encephalitis” [Title/Abstract] OR “tick borne encephalitis” [Title/Abstract] OR “tick-borne encephalitis virus” [Title/Abstract] OR “tick borne encephalitis virus” [Title/Abstract] OR “TBE” [Title/Abstract] OR “TBEV” [Title/Abstract] OR “Encephalitis, Tick-Borne”[Mesh] OR “Encephalitis Viruses, Tick-Borne”[MeSH] OR “Tick borne encephalit*” [Title/abstract] OR “tickborne encephalit*”[Title/Abstract] OR “tick encephalit*”[Title/Abstract] OR “tick borne meningoencephalitis”[Title/Abstract] OR “tickborne meningoencephalitis”[Title/Abstract] OR “tick-borne virus encephalit*”[Title/Abstract] OR “tickborne virus encephalit*”[Title/Abstract] OR “TBE virus*”[Title/abstract] OR TBEV[Title/Abstract] OR “Encephalitis Viruses, Tick-Borne” [Title/Abstract] OR “Encephalitis Viruses, Tick Borne” [Title/Abstract] OR “Encephalitis Virus, Tick-Borne”[Title/Abstract] OR “Encephalitis Virus, Tick Borne”[Title/Abstract] OR “Viruses, Tick-Borne Encephalitis”[Title/Abstract] OR “central european encephalit*”[Title/Abstract] OR “tick-borne viral encephalitis”[Title/Abstract] OR “tickborne viral encephalitis”[Title/Abstract] OR “tick-born encephalit*”[Title/Abstract] OR “Far Eastern Russian Encephalit*”[Title/Abstract] OR “Russian Spring-Summer Encephalit*”[Title/Abstract] OR “Louping Ill Encephalit*”[Title/Abstract] OR “Powassan Encephalit*”[Text Word] OR “Powassan Virus Diseas*”[Title/Abstract] OR “FSME” [Title/Abstract] OR “Frühsommer Meningoencephalitis” [Title/Abstract])

Search terms for Seroprevalence:

- AND – (“seroprevalence” [Title/Abstract] OR “human seroprevalence” [Title/Abstract] OR “immunity” [Title/Abstract] OR “seroprevalence immunity” [Title/Abstract] OR “natural immunity” [Title/Abstract] OR “vaccine immunity” [Title/Abstract] OR “vaccination immunity” [Title/Abstract] OR “natural vs vaccine immunity” [Title/Abstract] OR “natural vs. vaccine immunity” [Title/Abstract] OR “natural versus vaccine immunity” [Title/Abstract] OR “seropersistence” [Title/Abstract])
- OR/AND - (“antibody” [Title/Abstract] OR “antibody response” [Title/Abstract] OR “antibodies” [Title/Abstract] OR “duration” [Title/Abstract] OR “antibody duration” [Title/Abstract] OR “serology” [Title/Abstract] OR “persistence” [Title/Abstract] OR “antibody persistence” [Title/Abstract] OR “waning” [Title/Abstract] OR “waning antibody” [Title/Abstract] OR “waning antibody response” [Title/Abstract])
- OR/AND - ("Seroepidemiologic Studies"[Mesh] OR Seroepidemiolog*[Title/Abstract] OR "seroprevalen*"[Title/Abstract] OR “sero-prevalen*”[Title/Abstract] OR “serologic prevalen*”[Title/Abstract] OR “serological prevalen*”[Title/Abstract] OR “serology prevalen*”[Title/Abstract] OR “seropositiv*”[Title/Abstract] OR "Seroconversion"[Mesh] OR seroconversion*[Title/Abstract] OR “sero-conversion*”[Title/Abstract] OR "serosurvey*"[Title/Abstract] OR “sero survey*”[Title/Abstract] OR "Antibodies"[Mesh:NoExp] OR "antibod*"[Title/Abstract] OR “immune factor*”[Title/Abstract])
